# Rotating with the brakes on and other unresolved features of the vacuolar ATPase

**DOI:** 10.1042/BST20160043

**Published:** 2016-06-09

**Authors:** Shaun Rawson, Michael A. Harrison, Stephen P. Muench

**Affiliations:** * School of Biomedical Sciences, Faculty of Biological Sciences, University of Leeds, Leeds LS2 9JT, U.K.

**Keywords:** cryo-EM, molecular motor, rotary ATPase, vacuolar ATPase

## Abstract

The rotary ATPase family comprises the ATP synthase (F-ATPase), vacuolar ATPase (V-ATPase) and archaeal ATPase (A-ATPase). These either predominantly utilize a proton gradient for ATP synthesis or use ATP to produce a proton gradient, driving secondary transport and acidifying organelles. With advances in EM has come a significant increase in our understanding of the rotary ATPase family. Following the sub nm resolution reconstructions of both the F- and V-ATPases, the secondary structure organization of the elusive subunit *a* has now been resolved, revealing a novel helical arrangement. Despite these significant developments in our understanding of the rotary ATPases, there are still a number of unresolved questions about the mechanism, regulation and overall architecture, which this mini-review aims to highlight and discuss.

## Introduction

**Figure 1:**
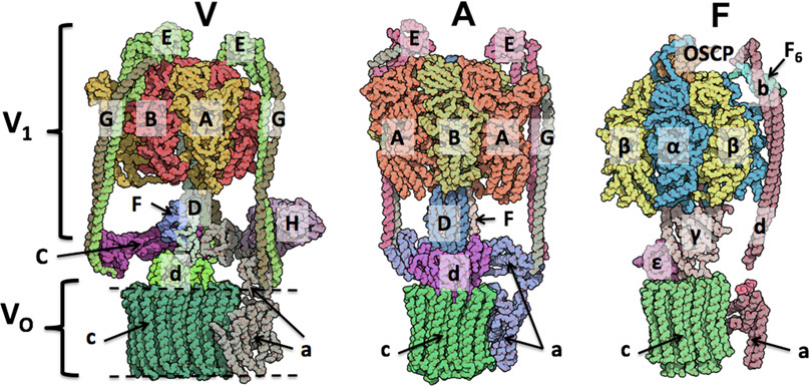
Schematic view of the rotary ATPase family Subunit organization in the rotary V-/A-/F-ATPase families. Membrane is indicated by dashed line.

The rotary ATPase families are membrane-bound molecular motors which act either by hydrolysing ATP to generate a proton gradient or by utilizing an existing proton gradient to energize ATP synthesis. The family consists of the ATP synthase (F-ATPase), vacuolar ATPase (V-ATPase) and archaeal ATPase (A-ATPase). A common feature of the rotary ATPase family is the coupling of a membrane-bound proton-translocating domain (V_O_) and a soluble, ATP hydrolysing/ synthesizing motor (Figure 1). Although the core motors of each member of the family share a high degree of similarity, they possess varying degrees of extra complexity. This is indicated by the number of static peripheral stalks (that make up the ‘stator’ of the motor) they possess which may reflect the differing degrees of regulation they each require [1]. The best studied system, the F-ATPase, is the simplest of the family, possessing only one stator stalk. It primarily uses the proton gradient and subsequent flow of H^+^ through the F_o_ domain to generate torque and drive ATP synthesis in the F_1_ region. Recent EM and crystallography studies have shown the interface where proton translocation occurs between the rotating *c*-ring and the static subunit *a* has a novel architecture with two horizontal helices parallel to the plane of the membrane and embedded within it [2–4]*.* At this interface the proton enters one side of a half-channel within *a*, is picked up by a conserved glutamate residue on the *c*-ring and following a full revolution of the rotor, is then moved into the half-channel on the opposite side. The second member of the ATPase family is the A-ATPase, which differs in organization to the F-type in having two stator stalks rather than one. Each of these stalks is made of just two coiled-coils rather than the multi-polypeptide stator found in the F-ATPase. Some members of the A-ATPase family also function through the use of sodium as a coupling ion [5].

This review focuses on the V-ATPase, which acts via the hydrolysis of ATP to drive proton translocation across a membrane. The V-ATPase has the same overall architecture as the other ATPase family members consisting of the soluble ATP hydrolysing domain (V_1_) and V_O_ (Figure 1). V_1_ consists of three AB repeats where ATP hydrolysis takes place, subunits D and F make up the central rotor axle and three stator stalks consisting of subunits E and G. The three stators are linked by a series of ‘collar’ subunits which include subunit C, H and the soluble domain of subunit *a*. The membrane embedded V_O_ domain consists of the *c*-ring, made up of a variable number of *c* subunits, subunit *d* and the remainder of subunit *a*. The V-ATPase plays an essential role in several physiological processes, including cell homoeostasis and signalling [6], and therefore is found in all eukaryotic cells [7]. It is thus unsurprising that the complex is implicated in a variety of disease states [8], for example its role in bone resorption through acid extrusion means that it is involved in both osteoporosis and osteopetrosis [9]. The V-ATPase has also been linked to cancer invasiveness and has been studied as a target for cancer therapy [10–12].

Recently, the sub nm resolution EM structures of the V-ATPase from both yeast and the higher eukaryote *Manduca sexta* [13,14] have provided unprecedented detail into its structure and mechanism. In particular, the structures from yeast show the organization of subunit *a*, in the form of highly tilted horizontal helices similar to the arrangement recently observed in F-ATPase from *Polytomella* sp [2]. Despite the improved resolution of these structures, the primary structure of subunit *a* cannot be unambiguously assigned in the map hence, the connectivity between helices is still uncertain. The yeast V-ATPase has been solved in three distinct rotational states, permitting the conformational changes that accommodate ATP hydrolysis to be observed.

Despite the great advances that have been made in our structural and biochemical understanding of the rotary ATPase family, a number of unresolved questions remain, some of which this review aims to address. For example the manner in which isolated or disassociated domains of the complex are silenced, the effect of direct linkage between the stator and rotor domains acting as a ‘break’ and the role and location of subunits *e* and Ac45 within the complex.

### Mechanism

Through a combination of biochemical analysis, X-ray crystallography and EM, a number of defined catalytic states of the V-ATPase have been trapped which are consistent with the ‘Boyer mechanism’ [15–17]. Key to the rotation of the central axle is the movement of the lever arms at the base of the AB catalytic dimer, induced through ATP hydrolysis and the subsequent release of ADP and P_i_. The central rotor axle is stabilized at the top of V_1_ by a series of highly conserved loops that display alternative positive and negative charges (a feature conserved in the F- and A-ATPase families) and it is tempting to hypothesize that these act as a frictionless electrostatic bearing through a series of attracting and repelling charges acting on the charged axle [14,18]. To our knowledge there is no report of any mutagenesis on these residues, which could provide new insights into this poorly characterized highly conserved feature within the rotary ATPase family.

**Figure 2:**
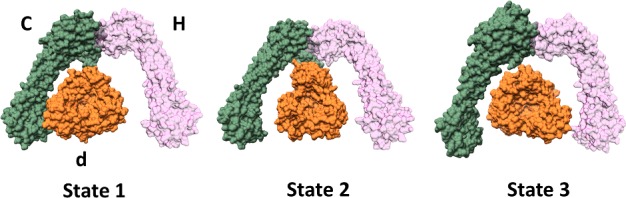
Subunit interactions about the collar region of the V-ATPase in 3 different conformational states Surface representations of collar subunits based on yeast cryo-EM structures for three rotational states [protein data bank (PDB): 3J9T, 3J9U, 3J9V] showing interactions between rotor subunit *d* (orange) and collar subunits C and H (green and pink). Shown looking down from V_1_ domain towards membrane.

Implicit within the rotary mechanism is the need for the central rotor axle to couple with the *c*-ring with no interactions to the static stator complex. It is clear that within both the yeast and *M. sexta* systems this is not the case with significant contact made between subunits *d* and C, which would be analogous to applying the brakes to the rotary mechanism (Figure 2) [13,14,19]. The removal of this linkage has been shown to increase the flexibility of the system [20]. Moreover, it was hypothesized that this may act in a ratchet-like mechanism such that the conformational changes which accommodate ATP binding and hydrolysis not only drive rotation but release the steric hindrance, allowing forward rotation [20]. In the absence of any ATP hydrolysis, the axle is unable to rotate either forwards or in reverse through steric hindrance of this ‘brake-like’ mechanism, silencing proton leakage and preventing futile backwards rotation of the rotor.

Analysis of the different catalytic states shows significant movement of the EG stators [13,21], although their fundamental shape is dictated not by the occupancy of the ATP-binding site in the AB domain, but by interactions with the different subunits within the collar region [14]. Interestingly for both the *M. sexta* and yeast complexes there is an apparent ‘resting state’ into which a predominant population of V-ATPase exist, (approximately 48% within the yeast system [13]). These states also coincide with the level of interaction between subunit *d* and subunits C and H. Within yeast the largest to smallest interfaces have approximately 48%, 36% and 16% of particles associated with them, respectively. This suggests that the interface may potentially provide stability within the complex, although the exact purpose is unknown. It may be that this asymmetry in population is related to regulation, as it is known that addition of ATP induces dissociation of the complex [22], so it is possible that the detachment of V_1_ from V_o_ can only occur in certain rotational states and thus is mediated by the stability of this interface.

### Regulation

The ability of the V-ATPase to consume significant cellular resources of ATP [22,23], requires a regulatory mechanism to avoid futile ATP turnover. One proposed mechanism is through the dissociation of V_1_ from V_O_, as shown in yeast and *M. sexta* [24,25]. However, recent *in vivo* experiments have suggested a more subtle rearrangement of V_1_ from V_o_ rather than complete separation [26]. Moreover, to date evidence for this being a ubiquitous mechanism also remains elusive with other species failing to show the same dissociation behaviour, although there is some evidence in mammalian cells for amino acid modulated reassembly [27]. Furthermore, although the A_1_ domain from the A-ATPase has been well studied, to our knowledge it has not yet been shown to dissociate in a similar manner to that of the V-ATPase under physiological conditions. In addition to the need for regulation of the mature complex, it is also vital to regulate the isolated hydrolysing domains of the complex along the assembly pathway. This may be regulated in an alternative manner or share a common method of regulation with the dissociated V_1_/A_1_. Therefore, it is possible that, although informative, studies that are carried out on the isolated A_1_ domain may be more relevant to a stable assembly intermediate rather than representative of a dissociated A-ATPase complex, whereas V_1_ can be isolated after controlled dissociation [28].

Within the V-ATPase, subunits C and H have been implicated in the regulatory process with the former responding to cellular signals (likely phosphorylation [29,30]), triggering dissociation and then later halting rotation of the central axle through steric hindrance [31]. The A-ATPase lacks any homologues for these subunits and so the triggers for dissociation and/or silencing of ATP turnover are unknown if indeed present. With regards to ATP silencing, there is evidence that subunit H undergoes an approximately 120° rotation about the EG stator to interact with the central rotor axle [31,32]. The interfaces involved within this interaction are yet to be resolved with an approximately 25 Å (1 Å=0.1 nm) EM reconstruction of the isolated V_1_ domain from *M. sexta* failing to show any interaction despite the H subunit being associated with the complex as shown through biochemical and negative stain analysis [28]. The lack of strong density for subunit H may represent a high degree of flexibility or an artefact caused through the modest resolution. It has also been suggested that subunit F, which sits at the base of the rotor axle, may play a role in silencing ATP hydrolysis in the isolated V_1_ and A_1_ domains [33,34]. As the F subunit is common to both the V- and A-ATPase, it is possible that this forms the basis of a common mode of silencing during the process of complex assembly.

### The role of subunits *e* and ac45

**Figure 3:**
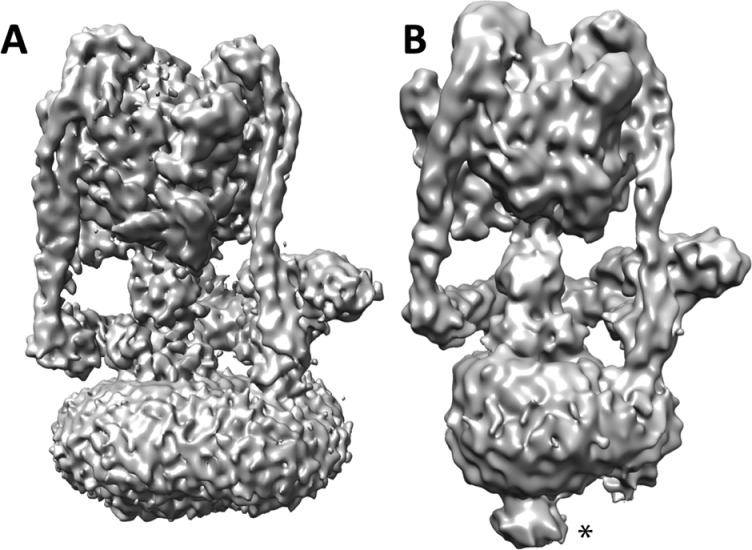
Structural comparison between yeast and *M. sexta* V-ATPase A comparison of the single particle 3D reconstruction of the V-ATPase from yeast (**A**) and *M. sexta* (**B**). The protrusion of density at the base of V_o_ in the *M. sexta* map is highlighted by a star. Figures reproduced from [13]: Zhao, J., Benlekbir, S. and Rubinstein, J.L. (2015) Electron cryomicroscopy observation of rotational states in a eukaryotic V-ATPase. Nature **521**, 241–245 and [14]: Rawson, S., Phillips, C., Huss, M., Tiburcy, F., Wieczorek, H., Trinick, J., Harrison, M.A. and Muench, S.P. (2015) Structure of the vacuolar H(+)-ATPase rotary motor reveals new mechanistic insights. Structure **23**, 461–471.

The least characterized subunit within the V-ATPase complex is subunit *e*. It has been shown to be heavily glycosylated [35] and is required for accurate assembly of the *c*-ring [36–38]. However, its role, if any, in V-ATPase mechanism and/or structure is unknown. Within yeast, it has been shown that subunit *e* is absent from the complex after purification [39]. However, for *M. sexta,* the *e* subunit has been identified as part of the purified complex [35]. Moreover, the potent and selective V-ATPase inhibitor Pea albumin toxin 1b (PA1b) was shown to bind subunit *e* with the corresponding position in the EM map at the base of the V-ATPase [40]. There is a clear distinction at the base of the V-ATPase, between yeast and *M. sexta,* with the latter, which contains subunit *e,* having a protrusion that is partially glycosylated at the base of V_o_ [14] (Figure 3). For the yeast enzyme, which lacks subunit *e* in the purified complex, no protrusion is seen [13]. Therefore, we believe that subunit *e* is located at the base of the V-ATPase, although its function and its precise location are yet to be resolved. This is important as subunit *e* may be used as a target site for selective inhibitors as it is differentially observed across species. Thus, it would be valuable to study the relationship between organisms possessing the *e* subunit within the complex and those where it is more transient in order to discern the rationale behind the retention and thus the function of this mysterious subunit.

Previous studies have suggested the accessory protein Ac45 is responsible for the density at the base of the V-ATPase since it is absent from yeast, along with the protrusion at the base of V_o_ [41]. However, despite extensive MS and gel analysis Ac45 cannot be identified within the *M. sexta* V-ATPase preparation [14], which also contains a similar protrusion at the base of V_o_. Therefore, it seems logical to conclude that the density at the base of V_o_ is linked to subunit *e* and not Ac45. The role for Ac45 and its association with the V-ATPase is still poorly understood although there is growing evidence that it plays a role in localization rather than mechanism [42,43].

## Conclusions

Although there have been several major advances recently in our understanding of the structure and function of the V-ATPase and rotary ATPases in general, there are still several unanswered questions. Through understanding these systems in more detail we are beginning to see subtle differences between organisms, such as the presence or absence of subunit *e* and Ac45, which may call into question the relevance of model systems currently in use. Mechanistically, there are still several challenges to overcome such as the manner of regulation and dissociation within the V-ATPase and how this differs across the ATPase family as a whole, and also the safeguards in place to prevent backwards rotation. Additionally, the precise mechanism of proton translocation is still unknown with higher resolution structural studies still required to view this process in detail, whereas dynamic structural studies may enable functional and mechanistic detail to be linked to structural changes rather than the static snapshots currently available.

## Abbreviations

A-ATPase: archaeal ATPase
F-ATPase: ATP synthase
PA1b: Pea albumin toxin 1b
PDB: protein data bank
V-ATPase: vacuolar ATPase
V_O_: membrane-bound proton-translocating domain
V_1_: ATP hydrolysing domain
